# Swearing as a performance aid: promising evidence, premature promotion

**DOI:** 10.3389/fspor.2026.1930904

**Published:** 2026-10-30

**Authors:** Nicholas B. Washmuth, Lia Jiannine, Christopher G. Ballmann

**Affiliations:** 1 Department of Psychology, The University of Alabama in Huntsville, Huntsville, AL, United States; 2 Department of Health and Human Performance, Nova Southeastern University, Fort Lauderdale, FL, United States; 3 Department of Human Studies, The University of Alabama at Birmingham, Birmingham, AL, United States

**Keywords:** ergogenic, exercise, physical performance, profanity, Rogers' diffusion of innovations

## Abstract

Swearing has recently emerged as a promising strategy for improving performance on short, intense physical tasks. Controlled experimental studies have shown that swearing can improve strength, anaerobic power, muscle endurance, and explosive performance, although the underlying psychological and physiological mechanisms remain poorly understood. Due to the inappropriate nature of swearing in social settings, the translation of swearing’s controlled experimental effects to the real world requires mindful evaluation. This perspective paper uses Rogers’ Diffusion of Innovations framework (relative advantage, compatibility, complexity, trialability, and observability) to provide a theory-informed evaluation to determine whether swearing should be promoted in real-world settings. Although swearing is cost-free, readily available, and easy to test, researchers have tested its effects only in controlled experimental contexts. Because the effects of swearing are context-dependent, its impact on performance likely varies by setting, audience, demographics, and cultural norms. We conclude that broad promotion is premature, but swearing warrants continued investigation as a performance-enhancing strategy with potential practical value.

## 1 Introduction

In the 1993 blockbuster movie *Jurassic Park*, the chaos theorist, character Ian Malcolm, warns that the “scientists were so preoccupied with whether or not they could, they didn’t stop to think if they should” create a wildlife park of de-extinct dinosaurs (00:30:44) (28). Although fictional, this quote highlights an important translational problem in science: demonstrating that an innovation has a significant effect in controlled settings does not necessarily indicate it should be adapted in practice. This *Jurassic Park* quote captures the concerns surrounding the emerging evidence on swearing and physical performance. Across multiple laboratory studies, swearing has been found to improve performance in short, intense physical tasks (19, 25, 33–35), providing growing evidence for an ergogenic effect. Thus, discussions have progressed from asking whether swearing can improve performance to asking whether we should promote swearing in real-world settings.

The translation of laboratory effects of swearing into real-world recommendations is not automatic, due to both laboratory research design constraints (e.g., internal validity, convenience sampling) and the context-dependent nature of swearing (18). Rogers’ (26) Diffusion of Innovations framework is useful here because it focuses on the broader conditions under which swearing would be used Rogers. (26) uses five criteria to guide the decision to adopt an innovative idea: relative advantage, compatibility, complexity, trialability, and observability. Innovations perceived as better than what they replace, more compatible with current norms, easy to use, easy to test, and having visible results tend to be adopted more easily in real-world settings (27).

The translational questions of swearing’s implementation in real-world settings are well-suited to be evaluated through Rogers’ (26) Diffusion of Innovation theory (13). Stephens et al. (34, p. 1) describe swearing as “a cheap, readily available intervention that appears to encourage us to ‘not hold back’ and instead ‘go for it’ a little more, with positive benefits on physical performance.” Additionally, although evidence on swearing’s ergogenic effect is growing, the available research has originated from a relatively small research network and has relied on convenience sampling of young adults, making the generalizability of these findings to other populations and naturalistic settings unclear. Therefore, swearing’s real-world utility depends not only on whether it works, but on whether people think it is appropriate, safe, worth trying, and beneficial. The overall purpose of this paper is to use Rogers’ five criteria of innovation to provide a theory-informed assessment of potential barriers and facilitators to implementing swearing as a strategy to enhance physical performance in real-world settings. The recommendations regarding the practical application of swearing will be informed not only by its adoption potential, but also by the quality, relevance, and limitations of the existing evidence supporting its effectiveness. Furthermore, this framework will be used to identify gaps in the literature and prioritize a research agenda aimed at addressing the limitations of swearing as a performance-enhancing strategy.

### 1.1 Background: From taboo speech to performance tool

Swearing, or the use of potentially offensive taboo words, can be considered inappropriate or unacceptable in any given social context (6). Due to the pressures to conform to societal norms, most individuals inhibit their use of swearing, leading to swearing’s ability to elicit unique physiological, psychological, and social effects (31). The initial investigation into swearing’s ergogenic effects occurred in 2018, when Stephens et al. (35) conducted experiments examining swearing’s influence on the Wingate Anaerobic Test (WAnT) and grip strength. Swearing resulted in a 4.5% increase in power during the WAnT and an 8% increase in grip strength, as compared to repeating a neutral word. Stephens et al. (33) conducted a study to replicate and extend their previous findings. In their replication study, swearing was found to improve grip strength by 8%. Another experiment by Stephens et al. (33) examined the effect of swearing on a chair push-up task, a muscle endurance task requiring participants to raise their bodies and support their body weight on their hands and arms against the chair seat for as long as possible. Participants were able to hold this chair push-up position for 10% longer while swearing, as compared to repeating a neutral word. Jiannine and Antonio (19) investigated the effects of swearing on grip strength, push-ups to fatigue, and wall sit and plank exercise time to exhaustion. Swearing resulted in increased grip strength by 9%, push-ups to fatigue by 15%, wall-sit time to exhaustion by 22%, and plank exercise time to exhaustion by 12%. Stephens et al. (34) provided further evidence that swearing improves performance during the chair push-up task, with exploratory mediation analyses suggesting that psychological flow, self-confidence, and distraction may contribute to these effects. Most recently, Nguyen et al. (25) had participants shout a swear word or a neutral word as loudly as possible prior to completing an explosive bench press exercise and a repetitions-to-failure bench press exercise. While shouting a swear word did not influence bench press repetitions-to-failure, swearing did significantly improve explosive bench press performance. Since swearing out loud may not be feasible for all individuals in all situations, Washmuth et al. (37) investigated the effects of inner monologue swearing on physical performance. Inner monologue swearing did not influence grip strength, but participants were able to hold the wall sit and plank positions significantly longer while swearing (38).

Swearing appears to improve physical performance on short, intense tasks (e.g., WAnT, grip strength); however, its influence on longer-duration anaerobic tasks has been underexplored. The only investigation, to our knowledge, investigating swearing’s effect on aerobic endurance was by Ballmann et al. (4). In this study, participants completed a graded exercise test, consisting of 3-minute exercise stages on a treadmill, with each subsequent stage getting faster and steeper in incline. Participants advanced through the stages until volitional fatigue required them to stop. Swearing did not influence aerobic endurance compared to a neutral word; however, swearing did enhance and stabilize the participants’ mood during intense aerobic exercise (4).

## 2 Diffusion of innovation: relative advantage

Relative advantage reflects the perception that an innovation is better than another idea or practice (27). In this section, swearing is compared to the realistic alternatives of neutral word vocalization and music.

### 2.1 Advantage of swearing

Swearing appears to offer ergogenic benefits at no material cost. That is, swearing is a cost-free, equipment-free, and readily available strategy that has demonstrated improvements in laboratory measures of physical performance. Although some studies have reported moderate-to-large effect sizes, the practical significance of these effects for athletes and exercisers remains uncertain given the limited evidence from trained populations and naturalistic settings. Whether these laboratory improvements translate into meaningful benefits during training or athletic competition remains unknown. Future research involving trained athletes and ecologically valid performance outcomes is needed to establish the relative advantage of swearing’s ergogenic effects in real-world settings.

For example Stephens et al. (35) reported that swearing improved WAnT power by 4.5% (*n* = 29, *dz* = 0.62, *p* = 0.002) and grip strength by 8% (*n* = 52, *dz* = 0.72, *p* < 0.001), compared with repeated a neutral word. However, the participants in these studies were young adults and their training status was not clearly identified. More recently, Nguyen et al. (25) examined resistance-trained males (*n* = 11) and found that swearing improved explosive bench press velocity (*d* = 1.11, *p* = 0. 004) but not repetitions to failure, compared to a neutral word condition. Although these findings suggest an ergogenic effect of swearing, the relatively small samples, controlled laboratory settings, and variability in performance tasks make it difficult to interpret swearing’s practical significance.

### 2.2 Limitations of swearing

While evidence supports swearing as a strategy for improving physical performance, listening to self-selected music also shows consistent ergogenic benefits. Furthermore, listening to music has repeatedly been shown to have ergogenic benefits across a variety of exercise modes and, unlike swearing, includes beneficial effects on endurance-based tasks (3). As with swearing, the ergogenic effects of music also show moderate-to-large effect sizes (3, 20), suggesting its potential practical significance. It is unknown whether swearing offers greater, or comparable, ergogenic effects than listening to music, or whether the performance benefits of swearing and listening to music are additive, where both swearing and listening to music improve performance beyond either swearing or listening to music. Interestingly, Ballmann et al. (5) found that listening to music with censored lyrics led to a decrease in bench press performance, as compared to music with explicit lyrics. Considering the general ergogenic effects of music (3), it is plausible that swearing and listening to music have a synergistic effect on performance. However, this is speculative, and future research should directly examine the effects of swearing, listening to music, and a combination of swearing and listening to music on physical performance.

Swearing has been shown to improve physical performance compared to repeating a neutral word across controlled experimental studies, including both in-person and remotely supervised procedures, using convenience samples of young adults. It is unknown whether swearing offers any benefit compared to no-vocalization (i.e., silence). That is, we don’t know if both swearing and repeating a neutral word benefit performance, and swearing just does so to a greater degree, or if both swearing and repeating a neutral word decrease performance, and swearing just does so to a lesser degree. Including a no-vocalization condition would also clarify plausible alternative explanations for swearing’s ergogenic effects, such as expectancy effects, motivational self-talk, attentional cueing, and placebo-like responses. It is also unknown if the ergogenic effects will translate into real-world settings, where contextual and interpersonal factors may affect swearing’s impact (18). Real-world external validity has not yet been established and, given the distinction between laboratory performance measures and naturalistic competitive sport performance, future research is required to investigate the transferability of controlled experimental findings into ecologically valid settings. Lastly, current evidence supporting swearing’s ergogenic effects has primarily relied on convenience sampling of college students, which is prone to selection bias (1).

## 3 Diffusion of innovation: compatibility

Compatibility refers to the degree to which an innovation aligns with the values and prior experiences of the potential adopters (27). In this section, the prevalence and social norms of swearing are used to help determine the compatibility of promoting swearing in real-world settings to enhance physical performance.

### 3.1 The compatibility of swearing in real-world settings

The effects of swearing are context-dependent (18). Unlike other ergogenic aids, such as nutritional supplements and equipment, which can be evaluated on the instrument itself, swearing must be evaluated within the context in which it occurs. These contextual factors include the social context in which it occurs, the relationships and demographics of those involved in the swearing encounter, and the perceived intent behind the swearing. Swearing is highly prevalent in today’s society. Most people admit to swearing “sometimes” or “often” (6), 70% of adults hear others swear in public (17), most workers swear in the workplace (21), and only 9% of college students report “never” hearing a professor swear (12). Therefore, swearing is commonly experienced and likely expected by most individuals, even in more formal settings (e.g., classroom, workplace).

### 3.2 Factors limiting the compatibility of swearing in real-world settings

While swearing appears prevalent and expected in today’s society, there may be some social norms and expectations that impact the outcomes of swearing in naturalistic settings. Studies demonstrating swearing’s ergogenic effects have been conducted in controlled experimental conditions, including researcher-supervised remote testing (33), where participants swear only in the presence of a single researcher; however, the social context contributes to the effects of swearing. Individuals define themselves partly through membership in social groups (e.g., gender groups, occupational groups), and behavior is guided by norms associated with those group identities. When these norms and expectations are violated, one’s sense of identity becomes salient, and various behaviors, such as swearing, invite social judgment. For example, swearing is often less socially acceptable for women, as compared to men (6, 7, 30). It is possible that the impact of swearing on performance depends on both the gender of the swearer and the gender(s) of the audience, as both males and females report swearing more comfortably in same-gender interactions and hesitate to swear in mixed-gender groups (6). It is also unknown if the ages of those involved impact the effect of swearing in naturalistic settings. It is plausible that swearing by, or in the presence of, younger or older populations may blunt the ergogenic effects of swearing due to the violation of social norms related to profanity and age (18).

The lack of ecologically valid studies limits our understanding of swearing’s effects on physical performance in real-world settings. Swearing has been shown to elicit distinct biopsychosocial effects, both positive and negative, that are not easily achieved with other forms of language (31). Because swearing has been shown to produce positive social effects (29), under certain circumstances swearing may be compatible in real-world settings. For example, swearing is often perceived as banter and an expression of affection, thereby increasing solidarity between the swearer and the audience (9, 24). Fine and Corte (10) describe “obscenity factories” as organizations or places where swearing is part of a member’s identity, being both expected and treated as routine. Since locker rooms have been described as “obscenity factories” (10), swearing may be compatible in specific real-world competitive sports contexts. Furthermore, Cai et al. (8) discuss informal norms and rituals as practical strategies to foster a cohesive team in sport. One informal norm or ritual could include swearing among in-group team members, which could increase compatibility in this real-world context if it serves to increase cohesion and solidarity among team members. Conversely, since swearing, by definition, has a strong potential to cause offense, it is not surprising that swearing can also produce negative outcomes. Malik and Ryan (23) provide a useful framework for why swearing may not be compatible in real-world settings. Because swearing provokes an emotional response in the listener, it negatively influences how the listener thinks, feels, and performs (23). Thus, swearing has been described as a “cognitive imposition.” For example, MacKay et al. (22) found that taboo words capture attention; however, athletes may respond more slowly to coaching cues when the cue is accompanied by a swear word, because swearing is attention-grabbing and causes athletes to focus on the swear word rather than the coaching cue (2). Izquierdo and Anguera (16) highlight that effective coach-to-player communication tends to be supportive and inclusive. A coach swearing at a player may be perceived as unsupportive and alienating, thereby decreasing motivation and solidarity. Washmuth et al. (38) found no significant overall effect of externally delivered verbal encouragement with swearing on WAnT performance. However, participant sex emerged as a moderator, such that males demonstrated lower average WAnT power when receiving verbal encouragement with swearing, while females showed no significant difference. These findings support the “cognitive imposition” framework. Unlike previous studies examining self-generated swearing, these findings from Washmuth et al. (38) suggest that the effects of externally delivered swearing may depend on the recipient’s characteristics, although the underlying social mechanisms remain unclear. Additionally, swearers are often perceived as socially inept, untrustworthy, and incompetent (14, 40). Taken together, swearing may not be universally compatible across all real-world settings and contexts.

## 4 Diffusion of innovation: complexity

Complexity refers to the degree to which an innovation is perceived as difficult to understand and use (27). At face value, swearing is prevalent, suggesting individuals already know how to swear, and swearing requires no equipment, certification, or financial obligation. However, while swearing itself has low complexity, its implementation (e.g., dosage, word selection) has some nuance.

### 4.1 The low complexity of swearing in real-world settings

Swearing is a cost-free, readily available linguistic tool. It can be self-generated at any time, and does not require a coach, app, or equipment. These aspects make swearing an attractive ergogenic aid.

### 4.2 The high complexity of swearing in real-world settings

On the surface, swearing appears simple. However, its real-world use raises questions based on the available evidence. Previous research demonstrating swearing’s ergogenic effects has asked participants to self-select their swear word, asking participants to choose a swear word in response to “banging their head accidentally” or “accidentally stubbing their toe” (39). The most common words selected by participants are *fuck* and *shit* (19). Although these self-selected swear words provide an ergogenic effect, it is unknown whether milder forms of profanity (e.g., *hell*, *damn*) offer a similar benefit, as milder profanity may improve compatibility. Additionally, the dosage of swearing, that is, when and how frequently the swearing occurs, has not been consistent across the studies showing swearing’s ergogenic benefits. While this suggests that a variety of dosages can produce beneficial effects on physical performance, we don’t know whether dosage matters. For example, Stephens et al. (35) had participants swear for 10 s just prior to testing their grip strength, while Jiannine and Antonio (19) had participants swear during their grip strength task. With respect to the frequency of swearing, studies have asked participants to swear every 2 s (33), every 3 s (35), every 5 s (19), or “at a steady pace” (35). Most studies have asked participants to repeat their self-selected swear word at a normal speech volume (39), while Nguyen et al. (25) asked participants to shout their swear word as loud as possible just prior to performing a physical task.

The habituation phenomenon has not yet been explored in the context of swearing’s ergogenic effects; however, there appears to be a habituation effect of swearing in other contexts. Stephens and Umland (36) revealed habituation to swearing’s hypoalgesic effects, where those who swear more frequently in their daily lives don’t get the same pain-modulating effects compared to those who swear less frequently. Research should explore habituation patterns of swearing in the context of physical performance.

## 5 Diffusion of innovation: trialability

Trialability is the degree to which an innovation can be tested on a limited basis (27). This may be swearing’s most convincing of Rogers’ five criteria.

### 5.1 Swearing is highly trialable

Swearing is easy to test. Individuals can try it immediately, in private, and at no cost. Based on the available evidence, athletes and exercisers can compare swear-word and neutral-word conditions within the same training session. This high trialability is useful because it enables continuous testing and refinement of swearing as a performance-enhancing strategy. Individuals can also seamlessly adjust word selection, dosage, and physical performance tasks.

### 5.2 Limitations of trialability

Although swearing is easy to test, there are limits here. Implementing swearing in private is easy. Trying it in a public gym or team competition, with people around, may have some negative consequences. There may be a social cost, such as negative perceptions from the audience, if the swearing offends others or violates norms (18, 30).

## 6 Diffusion of innovation: observability

Observability refers to the extent to which the results of an innovation are visible to others (27). From our perspective, the observability of swearing’s ergogenic effects is limited.

### 6.1 Why observability is limited

Swearing’s ergogenic effects, while practically meaningful from a research perspective, are embedded in strenuous tasks where many factors influence the outcome. Someone may voice an expletive and perform better, but that improvement may not be obvious to an observer. It is likely that the profanity itself may be more apparent than the performance improvement. Therefore, if an observer notices the swearing more easily than the performance improvement, the swearer may be perceived as antisocial or uneducated, rather than viewing swearing as a purposeful aid for performance.

### 6.2 Potential mechanisms for swearing’s ergogenic effects

Although distinct from Rogers’ observability criteria, understanding the mechanistic underpinnings of the beneficial effects of swearing on performance may help identify the conditions and contexts where swearing improves performance. The original theory that motivated research assessing the effects of swearing on physical performance assumed a psychophysiological mechanism linked to autonomic arousal (33), supported by evidence that swearing produces a “fight or flight” (e.g., sympathetic activation) response (32). However, Stephens et al.’s (35) initial investigation into swearing’s ergogenic effects showed that swearing benefitted performance in the absence of psychophysiological arousal. This prompted a shift to investigate the psychological mechanism of state disinhibition.

The state disinhibition theory posits that swearing violates social norms and diminishes internal psychological barriers, thereby allowing individuals to not hold back and take full advantage of resources such as physical strength, attentional focus, and determination (33). Consistent with this explanation, Stephen et al. (34) found that psychological flow, distraction, and self-confidence, constructs linked to state disinhibition, were significant statistical mediators. While state disinhibition was found to be a significant mediator, it explained a modest 14% (*R*^2^ = 0.14) of the variance in the effect of swearing on physical performance (34), suggesting there are likely other mechanisms through which swearing influences performance. Another potential psychological mechanism is the affective regulatory function of self-talk. Self-talk in sport helps individuals regulate emotional states (11). Therefore, swearing, as a form of self-talk, in addition to the psychological mechanism of state disinhibition, may also modify affective states during demanding or stressful tasks.

Sympathetic activation remains a plausible mechanism behind swearing’s ergogenic effects. Although Stephens et al. (35) found improved physical performance with swearing without a significant difference in measures of the “fight or flight” response, it is possible that the autonomic measures utilized in those experiments were insufficiently sensitive to capture subtle changes in autonomic activation. For instance, Stephens et al. (35) had participants swear for 10 s just prior to testing grip strength, while skin conductance was calculated as the average reading for the combined vocalization and grip procedure (35). One possible explanation is that the swearing-induced changes in skin conductance during the 10-second vocalization period were obscured when averaged across the combined vocalization and grip strength task; however, this explanation remains speculative. Another possible mechanism explaining swearing’s ergogenic effects is swearing-induced hypoalgesia (39). Swearing’s ability to modulate pain has been observed across studies (15). Interestingly, Stephens et al. (35) examined the effect of swearing on physical performance while also assessing pain. While swearing increased grip strength, it did not reduce pain perception. However, swearing-induced hypoalgesia remains a plausible but unconfirmed mechanism. Although swearing didn’t change perceptions of pain during the grip strength task, it may be that the swearing rendered the pain and discomfort of the grip strength task more tolerable, thereby allowing a greater amount of force to be exerted while pain ratings remained stable.

It is also quite possible that the mechanism behind swearing’s beneficial effects on physical performance includes a combination of state disinhibition, sympathetic activation, and hypoalgesia. However, their individual and combined contributions remain uncertain and warrant further investigation.

## 7 Future research agenda

Across Rogers’ (26) five criteria for deciding whether to adopt an innovative idea, several unanswered questions limit the extent to which swearing can be promoted as a real-world performance-enhancing strategy. Does swearing offer an advantage over existing strategies? Does the ergogenic effect of swearing generalize to naturalistic settings? Do demographic and audience factors moderate the effects of swearing? How should swearing be dosed? What mechanism(s) explain swearing’s performance benefits? Specific research priorities are described in Table 1.

**Table 1:** Prioritized research agenda that will help answer the question, “Should we promote swearing in real-world settings to enhance physical performance?”.

Priority	Rogers’ (2003) (26) criteria	Research priority/question	Rationale
1	Compatibility	What is the effect of swearing on physical performance in naturalistic settings, under ecologically valid conditions (e.g., public fitness center, team sport competition)?	Current evidence is derived from controlled laboratory settings. Because this paper focuses on real-world promotion, establishing external validity is the most important next step.
2	Compatibility	Do demographic variables (e.g., age, gender, religiosity, personality) of swearer or the audience moderate swearing’s ergogenic effects?	Swearing is highly context-dependent, and social norms of demographic variables may influence outcomes.
3	Relative advantage	How does swearing’s ergogenic effect hold up against a no-vocalization (i.e., silent) condition?	Existing studies compare swearing with a neutral-word vocalization. A silent condition is necessary to determine whether both swearing and repeating a neutral word benefit performance, and swearing just does so to a greater degree, or if both swearing and repeating a neutral word decrease performance, and swearing just does so to a lesser degree.
4	Relative advantage	How does swearing compare with self-selected music as a performance-enhancing strategy, and are these effects additive?	Swearing may offer practical value as an alternative or adjunct to established ergogenic strategies, even without demonstrating superiority. Research should determine whether swearing and music provide comparable or additive benefits.
5	Complexity	What dosage (e.g., frequency, timing, volume, duration) of swearing optimizes its ergogenic effects?	Existing studies have used a variety of dosages. Establishing an optimal or effective dose would make implementation practically interpretable.
6	Complexity	Does the intensity of the swear word (e.g., *fuck* vs. *damn*) impact its ergogenic effect?	If milder swearing offers similar benefits, swearing may be easier to implement across settings without a social cost.
7	Mechanisms (outside of Rogers’ framework)	What mechanisms (e.g., state disinhibition, sympathetic activation, hypoalgesia) explain beneficial effects of swearing on physical performance?	Identifying underlying mechanism may clarify when and how swearing improves physical performance.
8	Complexity	Is there a habituation effect of swearing’s performance benefits, across minutes, days, or weeks? If so, is it possible to be re-sensitized to swearing after being habituated?	Habituation could impact long-term usefulness and when this strategy should be used.
9	Trialability	Are there alternatives to vocal swearing (e.g., inner monologue swearing, reading swear words) that may be more socially acceptable and that impact physical performance?	More socially acceptable approaches may allow individuals to experiment with swearing while minimizing social consequences.
10	Relative advantage	What is the effect of swearing on aerobic endurance (e.g., 2,000-meter row, 5-kilometer run)?	It is important to determine which physical performance tasks may be influenced with swearing.

The research priorities presented in Table 1 reflect the authors’ judgment, guided by Rogers’ framework, the limitations of the existing evidence, and the real-world relevance of each unanswered question. The authors assigned greater priority to questions that directly influence whether swearing should be promoted in real-world settings. For example, the authors prioritized establishing effectiveness under naturalistic conditions over determining whether swearing outperforms silence because the paper’s primary objective is to evaluate swearing’s real-world utility. The rankings in Table 1 reflect the authors’ judgment rather than a validated prioritization procedure.

## 8 Discussion

This perspective paper used Rogers’ five-criteria framework to organize a theory-informed assessment of potential barriers and facilitators to adopt and promote swearing as a strategy to enhance physical performance in real-world settings. Importantly, Rogers’ framework assesses adoption potential rather than directly establishing an intervention’s effectiveness or whether to promote it. Based on the limitations of the available evidence, it is premature to promote swearing as a real-world strategy to enhance performance. Currently, the evidence is limited to controlled experimental studies. It is unknown whether the ergogenic effects of swearing found in these controlled experimental studies can be generalized to naturalistic settings. Furthermore, it is unknown whether there is a social cost to the swearer (e.g., being perceived as uneducated) or the audience (e.g., being offended). However, the current evidence is strong enough to support the continued investigation into the generalizability of swearing’s ergogenic effects. Based on the current evidence, swearing may serve as a performance-enhancing strategy under certain conditions; however, the impact of privacy, social context, and cultural compatibility factors remain unknown.

### 8.1 Conclusion

The science of swearing and physical performance has progressed to the point where translational questions can no longer be avoided. Whether swearing can improve performance is only the beginning. The more important question is whether swearing should be promoted in real-world settings.

The primary contribution of this perspective paper is the application of Rogers’ (26) Diffusion of Innovations framework to move beyond summarizing the current evidence and instead address key translational questions. While previous reviews have primarily synthesized whether swearing improves physical performance (39), the present paper extends this literature by evaluating whether swearing can be used as a performance-enhancing strategy in real-world settings. Using Rogers’ (26) Diffusion of Innovations framework to organize potential adoption barriers, this paper concludes that swearing should not be broadly promoted, at least for now. Importantly, this theory-informed assessment also identified specific gaps in the current evidence that limit swearing’s readiness for adoption and promotion. These gaps were used to prioritize a research agenda (see Table 1).

Although we should resist the broad implementation of swearing in real-world settings, we should not dismiss its potential. Swearing may prove to be a readily available strategy to improve performance across a variety of contexts. But before swearing can be promoted, future research is required to determine when, how, if, where, and with whom swearing can enhance physical performance.

## Data Availability

The original contributions presented in the study are included in the article/Supplementary Material, further inquiries can be directed to the corresponding author.
